# Genetic diversity, tissue-specific expression, and functional analysis of the *ATP7A* gene in sheep

**DOI:** 10.3389/fgene.2023.1239979

**Published:** 2023-09-20

**Authors:** Hao Li, Xiaolong Du, Xinyue Li, Pingjie Feng, Mingxing Chu, Yi Jin, Zhangyuan Pan

**Affiliations:** ^1^ Engineering Research Center of North-East Cold Region Beef Cattle Science & Technology Innovation, Ministry of Education, College of Agriculture, Yanbian University, Yanji, China; ^2^ Key Laboratory of Animal Genetics, Breeding and Reproduction of Ministry of Agriculture and Rural Affairs, Institute of Animal Science, Chinese Academy of Agricultural Sciences, Beijing, China

**Keywords:** *ATP7A*, sheep, horn, tissue-specific, function

## Abstract

In humans, variation of the *ATP7A* gene may cause cranial exostosis, which is similar to “human horn,” but the function of the *ATP7A* gene in sheep is still unknown. Tissue expression patterns and potential functional loci analysis of the *ATP7A* gene could help understand its function in sheep horn. In this study, we first identified tissue, sex, breed, and species-specific expression of the *ATP7A* gene in sheep based on the RNA-sequencing (RNA-seq) data. Second, the potential functional sites of the *ATP7A* gene were analyzed by using the whole genome sequencing (WGS) data of 99 sheep from 10 breeds. Last, the allele-specific expression of the *ATP7A* gene was explored. Our result showed the *ATP7A* gene has significantly higher expression in the big horn than in the small horn, and the *ATP7A* gene has high expression in the horn and skin, suggesting that this gene may be related to the horn. The PCA results show that the region around the *ATP7A* can distinguish horned and hornless groups to some extent, further indicating that the *ATP7A* may be related to horns. When compared with other species, we find seven ruminate specific amino acid sites of the ATP7A protein, which can be important to the ruminate horn. By analyzing WGS, we found 6 SNP sites with significant differences in frequency in horned and hornless populations, and most of these variants are present in the intron. But we still find some potential functional sites, including three missenses, three synonymous mutations, and four Indels. Finally, by combining the RNA-seq and WGS functional loci results, we find three mutations that showed allele-specific expression between big and small horns. This study shows that the *ATP7A* gene in sheep may be related to horn size, and several potential functional sites we identified here can be useful molecular markers for sheep horn breeding.

## 1 Introduction

Sheep are an important economic resource for humans since they provide a variety of products including meat, fur, skin, and milk (Simoes et al., 2021; Zeder, 2008). Similar to other ruminants, sheep normally have horns which is a bony cranial extension on their craniums (Simon et al., 2022; Wang et al., 2019). Horns serve a dual purpose in the natural world, functioning as a means of self-defense against predators and a primary mechanism for intra-species competition for mates and territory (Buntgen et al., 2018; Nasoori, 2020). However, in the process of breeding, the existence of horns is easy to cause harm to livestock and personnel, which is unfavorable to animal husbandry production management and has great safety risks. At the same time, the removal of horns by artificial means is not only contrary to animal welfare but also increases the workload, and is inherently undesirable. Therefore, farmers invest a lot of effort in selecting hornless animal breeds. Using gene-editing technology to edit specific genes, cows can be hornless, so that calves do not have to go through the painful process of being burned off their horns after birth (Mueller et al., 2021; Wang et al., 2022). But the hornless mutation used in gene-editing breeding in sheep has not yet been confirmed.

The *ATP7A* gene is an important gene located on the X chromosome, and the ATP7A protein is widely involved in the transport of metal ions and is responsible for the regulation of copper ions (Bitter et al., 2022). The ATP7A protein is mainly distributed in tissues such as the liver and intestines (Gao et al., 2021; Telianidis et al., 2013). The protein is involved in the physiological processes of copper uptake, transport, and storage (Sudhahar et al., 2019), and is an essential component of many important enzymes such as copper-dependent enzymes and cytochrome C oxidases (Guthrie et al., 2020; Horn and Wittung-Stafshede, 2021). In recent years, more and more studies have shown that mutations in the *ATP7A* gene are closely related to the occurrence of many important diseases. The disease caused by the *ATP7A* gene defect in the human body is collectively called an *ATP7A* gene-related disorder, which is mainly manifested as a disorder of copper metabolism (Chen et al., 2020). Mutations in the *ATP7A* gene cause Menkes disease. Menkes is an infantile, fatal, inherited copper deficiency characterized by progressive neurological damage that eventually causes death. A mild form of Menkes disease is called occipital angle syndrome (OHS) (Moller et al., 2021; Yasmeen et al., 2014), which showed a symptom of exophytic bone warts (De Feyter et al., 2023) that is similar to the bony core of the horn. So we suspect *ATP7A* can have an important role in the ovine horn. However, currently, there is no research on the *ATP7A* gene in sheep yet. In this study, RNA-seq data was used to character the expression of the *ATP7A* gene in different tissues of sheep, for getting the basic function of the *ATP7A* gene. Then some horn-relative functional loci of the *ATP7A* gene have been explored by combining WGS data from 10 sheep populations with different horn types. This result will provide some valuable molecular makers in sheep horn breeding.

## 2 Materials and methods

### 2.1 Ethical statement

All experiments involving animals were ethically approved by the Animal Ethics Committee of the Institute of Animal Science, Chinese Academy of Agricultural Sciences (IAS-CAAS) (Beijing, China) on 28 April 2020 (No. IAS 2020-63).

### 2.2 Animal and sample collection

The samples used in the RNA-seq study were collected from Tibetan sheep in Dangxiong, Tibet, China. This population consists of four scurred (0–12 cm) and four spiral and horizontally extended horned (>12 cm, SHE) (Supplementary Table S1). The soft-horned tissue of these eight sheep was collected, placed in cryopreservation tubes, and stored in liquid nitrogen.

### 2.3 RNA extraction, library construction, and sequencing

The total RNA of tissues was extracted using the Trizol (Thermo Scientific, Wilmington, United States) kit. Electrophoresis using agarose gels at a concentration of 1% to detect RNA degradation and contamination. RNA purity testing was performed using the Kaio K550 (CAIO, Beijing) spectrophotometer. The Qubit^®^ RNA Detection Kit in the Qubit^®^ 2.0 Fluorometer (Life Technologies, California, United States) is used to determine RNA concentration. RNA nano 2100 assay kit from the Agilent Bioanalyzer 6000 System (Agilent Technologies California, United States) for RNA integrity testing.

Take 3 μg of total RNA to construct the library. First, Ribo-ZeroTM GoldKits (Epicentre, United States) was used to remove ribosomal RNA (rRNA). In addition, RNA sequencing libraries were generated according to the manufacturer’s instructions for the Illumina NEB Next Ultra Directional RNA Library Preparation Kit (NEB, Ipswich, United States). Sequencing was then performed on the Hiseq X (Illumina, San Diego, CA, United States) platform.

### 2.4 Sequencing data filtering, comparison, assembly and processing

We removed adaptors and low-quality reads using Trim Galore (v.0.6.7). We mapped clean reads to the ARS-UI_Ramb_v2.0 sheep reference genome using paired mapping modules of STAR (v.2.7.7a) with parameters of “--chimSegmentMin 10” and “--outFilterMismatchNmax 3” (Davenport et al., 2022; Dobin et al., 2013). A total of 8 high-quality RNA-seq clean data were obtained for subsequent analysis, with unique mapping reads > 85% and the number of clean reads > 20,000,000 (Supplementary Table S2). We then obtained TPM (Transcripts Per Kilobase of exon model per Million mapped reads) and FPKM (Fragments Per Kilobase of exon model per Million mapped fragments) of genes using StringTie (v.2.1.5) (Kovaka et al., 2019; Pertea et al., 2016; Pertea et al., 2015; Shumate et al., 2022), and extracted raw read counts of them with featureCounts (v.2.0.1) (Liao et al., 2013; Liao et al., 2014). We followed the recommended best practice pipeline in the genome analysis toolkit (GATK) (v.4.2.5.0) and used default settings to perform SNP site prediction (McKenna et al., 2010; Van der Auwera et al., 2013). We conducted allele-specific expression (ASE) analysis using the GATK ASEReadCounter tool. We used Subread_to_DEXSeq (https://github.com/vivekbhr/Subread_to_DEXSeq) of dexseq_prepare_annotation2.py script to format the annotation (GTF) file of the genome, then used load_SubreadOutput.R in Rstudio reads the formatted GTF file and the counts’ matrix output by featureCounts, constructs the DEXSeqDataSetFromFeatureCounts (dds) object, and performs exon difference analysis.

### 2.5 Analysis of the expression profile of the *ATP7A* gene

To explore the tissue, sex, breed, and species-specific expression of the *ATP7A* gene, we used the ggplot2 package of Rstudio to draw the specific expression based on RNA-seq data. The other sheep data was 3889 high-quality RNA seq sequencing obtained after downloading and filtering from NCBI (https://www.ncbi.nlm.nih.gov/) and EBI (https://www.ebi.ac.uk/) (Supplementary Table S3). The data for pigs (2,652 samples) (Supplementary Table S4), cows (4,501 samples) (Supplementary Table S5), and humans (9,810 samples) (Supplementary Table S6) were obtained from their GTEx project (Ardlie et al., 2015; CONSORTIUM, 2020; Liu et al., 2022; Teng et al., 2022). The pig normalized gene expression (TPM) data were obtained in http://piggtex.farmgtex.org/. The cattle TPM data were obtained in https://cgtex.roslin.ed.ac.uk/. The human TPM data were obtained in https://gtexportal.org/home/datasets. After downloading, the mean values were calculated separately for each tissue. We used T-test to calculate the significant difference between scurred and SHE. The Wilcoxon test was used to calculate the significant differences between ram and ewe (Supplementary Table S7). The significant differences between breeds were calculated using variance tests (Supplementary Table S8).

### 2.6 Analysis and three-dimensional structure prediction of the ATP7A protein

To investigate the differences in ATP7A protein among different species, we used MEGA (v.5.0) (Kumar et al., 2008) to analyze the homologous evolution tree. Amino acid differences of ATP7A protein were analyzed by the ClustalW algorithm. The amino acid sequences of the *ATP7A* gene in sheep and other species were obtained from the NCBI gene database (Supplementary Table S9). The 3D structure of the *ATP7A* gene (TrEMBL: W5QAF8) in sheep is predicated on the UniProt (https://www.uniprot.org). We predicted and labeled the key binding site of sheep ATP7A protein based on the characteristic sequence DKTGT of human P-type ATPase(Sazinsky et al., 2006). Based on the 7si3.1.A P-type Cu(+) transporter model, it is located in amino acids 1044–1048.

### 2.7 Whole genome sequencing analysis

To explore the potential functional sites of the *ATP7A* gene and its 10,000 upstream and downstream base pairs (bp) based on our previous WGS data (Pan et al., 2018), we used plink2 (v2.00a2.3) (Chang et al., 2015) and vcftools (0.1.16) (Danecek et al., 2011) to analyze the allele frequency of each breeds, and analyze the F-statistics (Fst) between horned and hornless breeds. We used the ggplot2 package of Rstudio to draw the principal component analysis (PCA) of allele frequency and the scatter plot of Fst. The data comes from 99 sheep from 10 breeds, including 6 Mongolian breeds: STH (Small Tail Han sheep), WZ (Wuzhumuqin sheep), T (Tan sheep), H (Hu sheep), CB (Cele Black sheep), and BY (Bayinbuluke sheep); 3 Tibetan breeds: VT (Valley Tibetan sheep), PT (Prairie Tibetan sheep), OL (Oula sheep) and a European-originated breed: AM (Australian Merino sheep). Among them, all H and AM have no horns (hornless breeds). Some of STH, T, CB, and BY have horns (horned breeds). VT, PT, and OL have horn (horned breeds).

## 3 Results

### 3.1 The expression of *ATP7A* in sheep with different horn types

**FIGURE 1:**
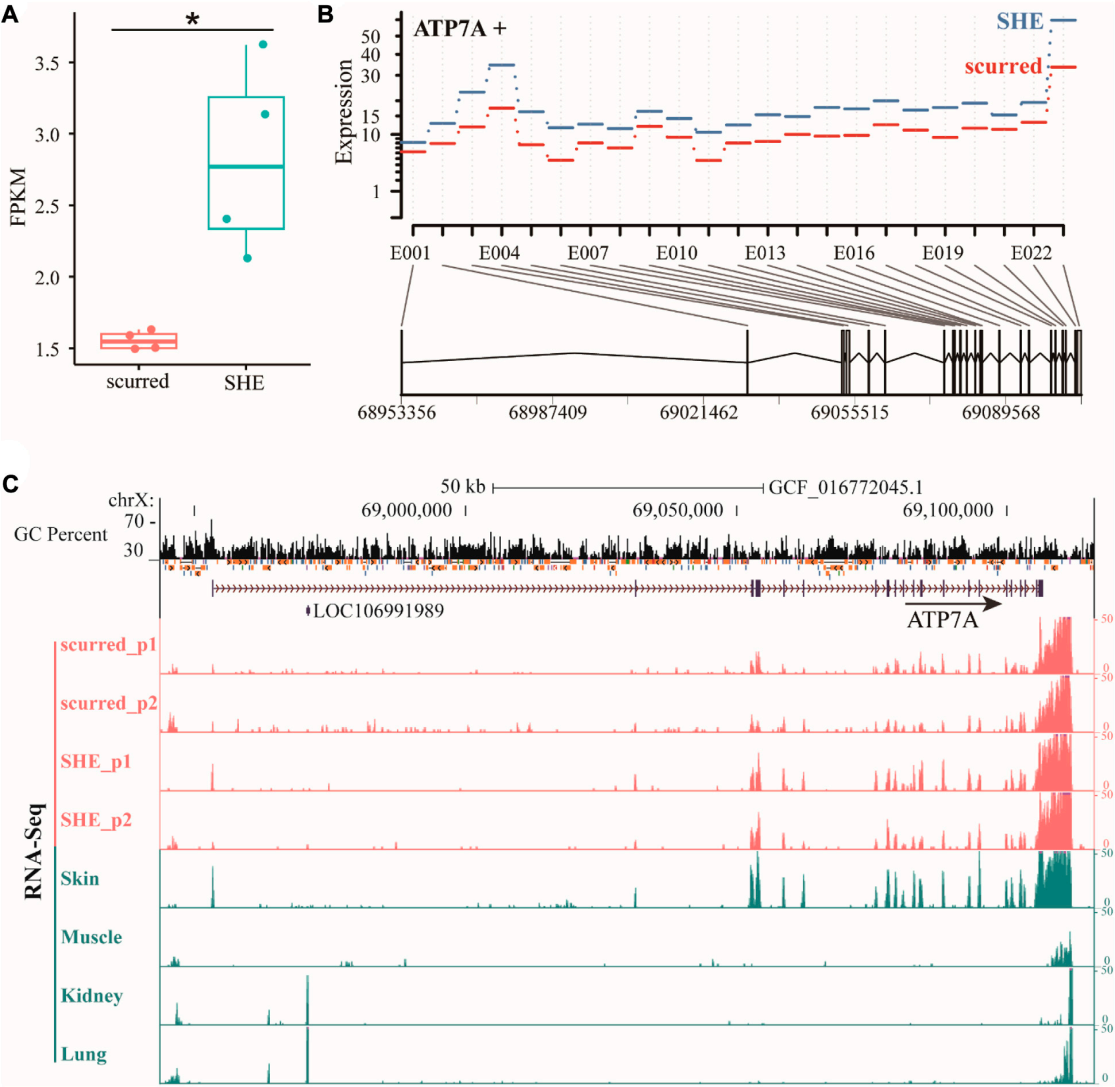
**(A)** Differential expression of *ATP7A* gene in scurred and SHE groups, the Expression is the fitted expression estimates from the glm regression, E001–E023 represents the number of Exon **(B)** differential expression of *ATP7A* gene exons in scurred and SHE groups **(C)** differential expression of *ATP7A* gene in horn tissues and other tissues.

The *ATP7A* (ATPase copper transporting alpha) gene is a protein-coding gene that belongs to the cation transport ATPase (P-type) (TC 3.A.3) family. It is part of the type IB subfamily. Based on gene annotation of ARS-UI_Ramb_v2.0 (GCF_016772045.1) version, *ATP7A* gene is located on the X chromosome and has two transcripts. The length of the above two transcripts is consistent, both are 153,239 bp. Compared to transcript XM_004022208.5, transcript XM_042242494.1 is missing exon 2. We will use transcript XM_004022208.5 to represent the ATP7A gene in this paper, which has the largest number of exons. From the RNA-seq results (Figure 1A), it can be seen that the FPKM of the *ATP7A* gene in the SHE group is significantly higher (*p* < 0.05) than in the scurred group. Exon expression results (Figure 1B) showed that the expression of exons of the *ATP7A* gene in the SHE group was higher than in the scurred group. We specifically checked the gene expression, GC percent, and repeat of the *ATP7A* gene (Figure 1C). We observed that the gene had readouts in the exons of each gene annotation, but there were still some expression readouts in regions without gene annotations, indicating that the gene still had some splicing without annotations. For example, there is a long Intron region between Exon 1 and 2. There were some expression readouts located this region in the scurred group and skin, fewer in the SHE group, almost none in muscles, kidneys, and lungs. The same situation exists between Exon 2 and 3. The expression of the *ATP7A* gene in horn tissues and skin tissues was higher, and in the SHE group is higher than the scurred group. *ATP7A* gene expression is usually low in muscles, kidneys, and lungs. This suggests that the *ATP7A* gene has some tissue specificity and may be related to horn function and epithelial function. Interestingly, we observed higher readings within about 5,000 bp (possibly poly-A tail) downstream of the *ATP7A* gene.

### 3.2 The tissue-specific function of the *ATP7A* gene in sheep

**FIGURE 2:**
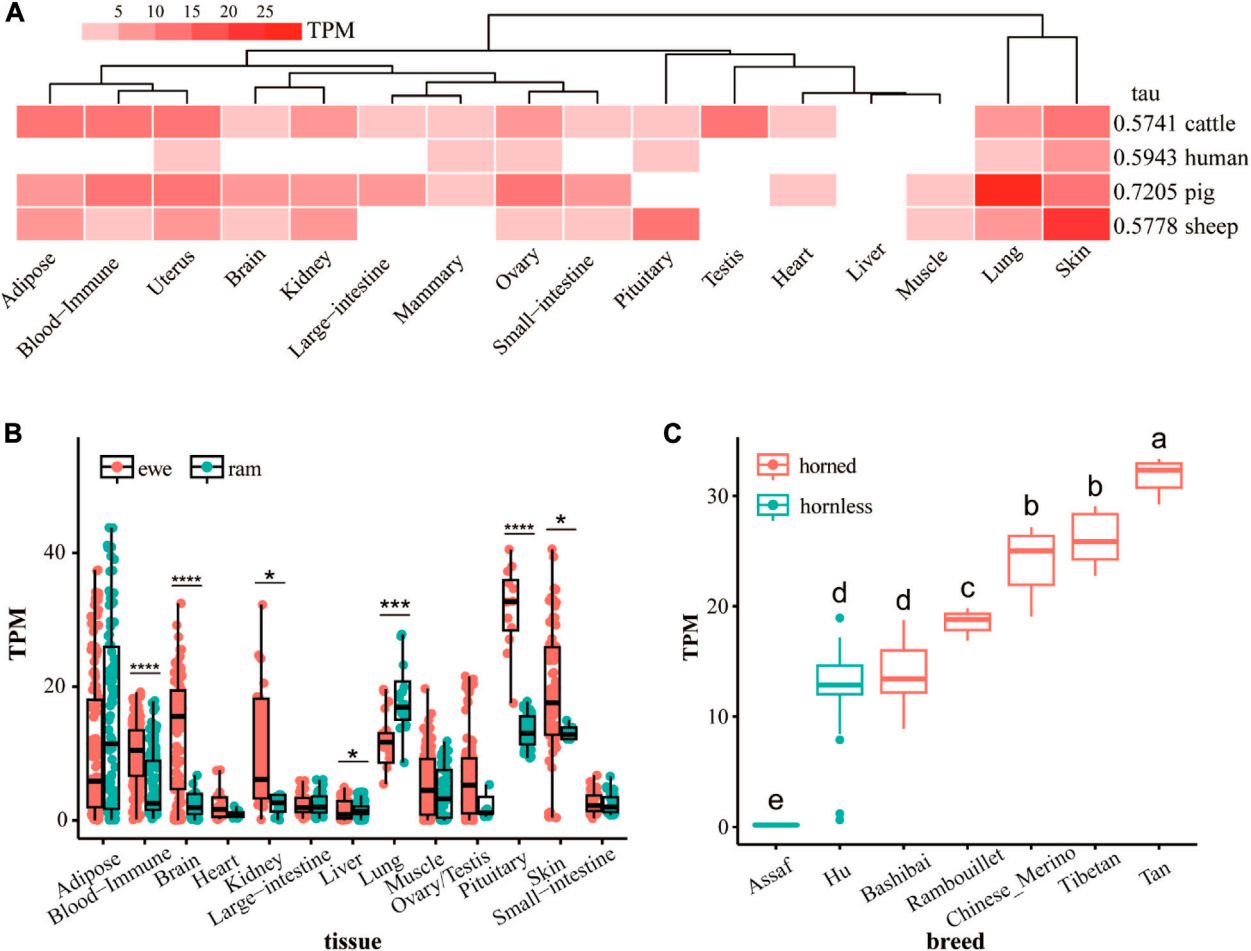
**(A)** The difference of RNA-seq expression of *ATP7A* gene in different species and tissues, the value is the mean of multiple replicates samples. **(B)** The difference of RNA-seq expression of *ATP7A* gene in tissues of rams and ewes **(C)** the difference of RNA-seq expression of *ATP7A* gene in skin tissues of horned and hornless sheep.

The tissue-specific heat map and tau values of the *ATP7A* gene for different species (Figure 2A) shows that the *ATP7A* gene has strong tissue specificity. The expression of the *ATP7A* gene in sheep skin tissue is much higher than in other tissues. At the same time, the gene is highly expressed in pig lung tissue. In humans and cattle, the tissue specificity of this gene is not obvious. Tissue-specific results for ewes and rams (Figure 2B) show sex differences in the expression of the *ATP7A* gene. The expression of this gene in the pituitary is the highest, and in the ewe is the most significantly higher (*p* < 0.0001) than the ram. Secondly, the gene is also highly expressed in skin, pituitary, adipose tissue, brain, and kidney. In the skin, the expression of the ewe is significantly higher than that of the ram (*p < 0.05*). The expression level of ram in the lung was extremely and significantly higher (*p* < 0.001) than that of the ewe. There were also significant differences between ewes and rams in blood immune, brain, kidney, and liver. The expression of the *ATP7A* gene in the skin of different breeds of sheep (Figure 2C) showed that the expression level of this gene is the lowest in Assaf sheep and the highest in Tan sheep. The expression of this gene in the hornless breed is significantly lower than that in the horned breed. As a breed mainly used for producing leather, although Hu sheep have no horns, they have thicker skin. This may be the reason for the high expression of the *ATP7A* gene in Hu sheep.

### 3.3 The potential function of ATP7A protein

**FIGURE 3:**
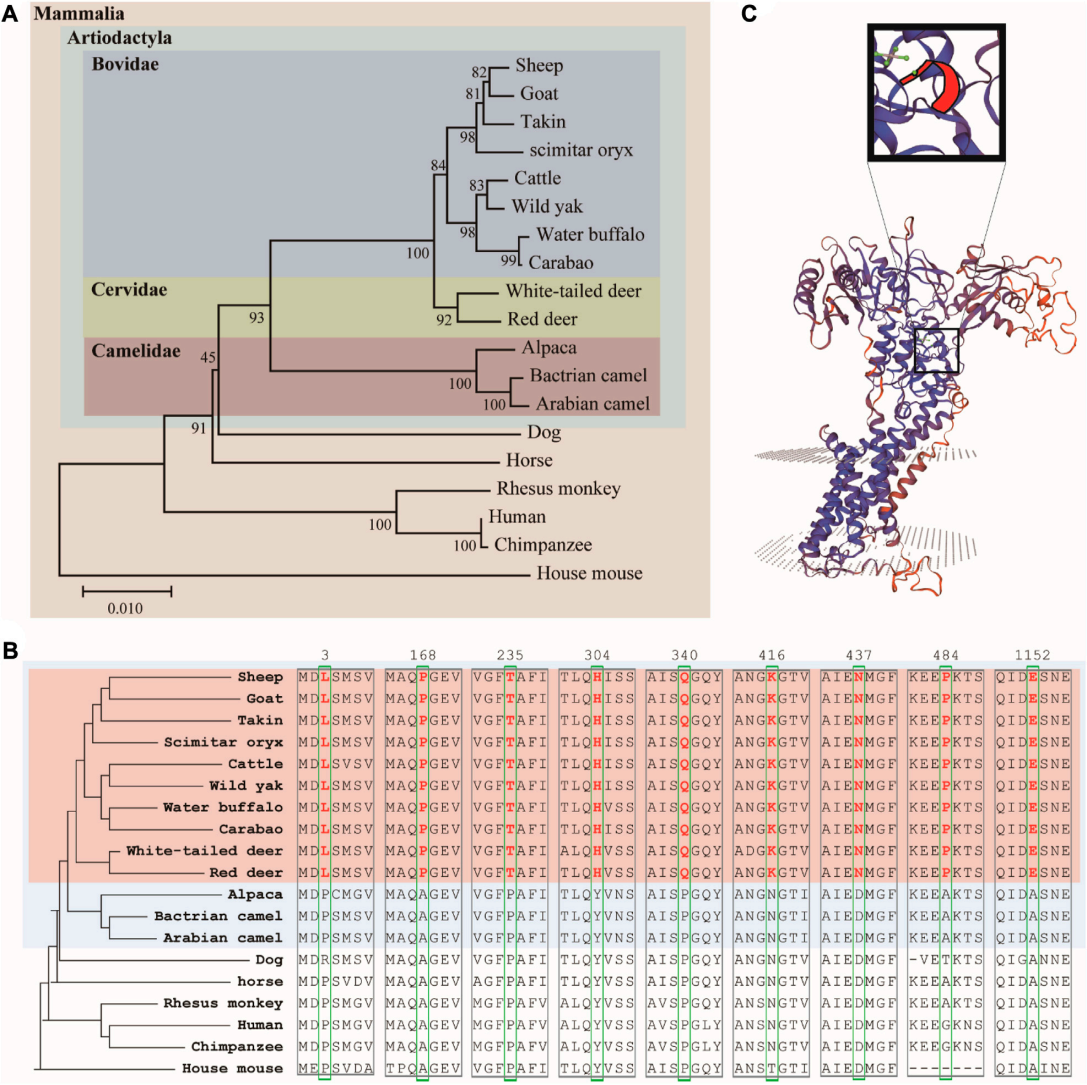
**(A)** Phylogenetic tree of ATP7A homologous protein sequences **(B)** the amino acid sites of seven ATP7A proteins unique to ruminants **(C)** three-dimensional structure and key positions of ATP7A protein.

We used MEGA to establish a phylogenetic tree of 19 animal ATP7A proteins using the adjacency method (Figure 3A). The results showed that Artiodactyla is clustered together, and Bovidae and Cervidae are closer (both have horn). At the same time, we found some interesting amino acid sites in the comparison results of amino acid sequences (Figure 3B). The 3, 168, 235, 304, 340, 416, 437, 484, and 1152 amino acids of the ATP7A protein in Bovidae and Cervidae (pink region) have specific amino acid sites, which are different from other animals. The above loci exist in Artiodactyla with horn, rather than in all Artiodactyla (blue region). These amino acid sites may be related to horn. Finally, we predicted the three-dimensional structure and key positions of the ATP7A protein (Figure 3C).

### 3.4 The potential function mutations in the *ATP7A* gene

**FIGURE 4:**
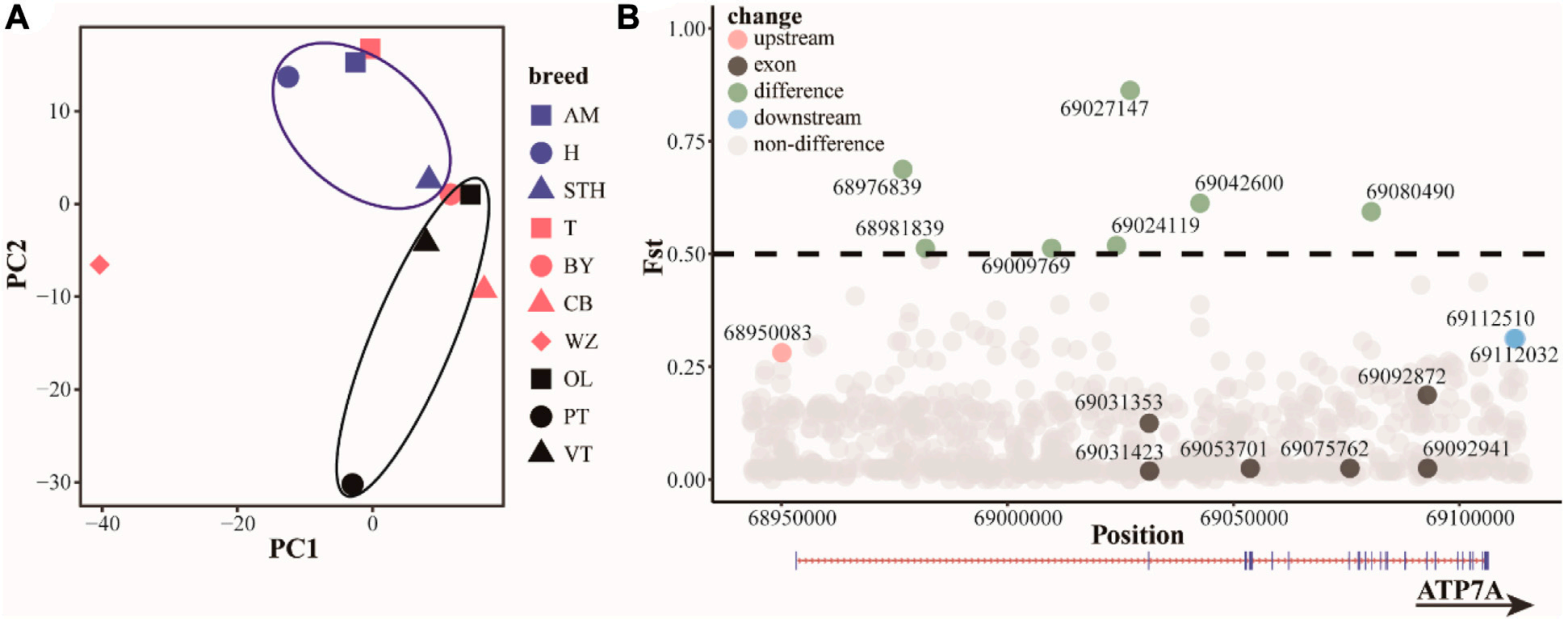
**(A)** Principal component analysis (PCA) of *ATP7A* region. Blue: hornless or partially hornless, red: most individuals horned, black: horned Tibetan breeds **(B)** differential distribution (Fst) of functional sites in the *ATP7A* region.

Based on the results of principal component analysis (PCA) (Figure 4A), hornless Australian merino sheep (AM) and Hu sheep (H) clustered together. Small-tailed sheep (STH) ewes have small horns or no horns, cluster in horned populations, and are relatively close to hornless populations. Among the Tibetan sheep breeds, the Prairie Tibetan sheep (PT) is far from the others, probably because the breed mostly has horns. This result indicates that although the functional sites in the *ATP7A* gene region cannot completely separate horned or hornless breeds, they can to some extent separate populations with horn differences, indicating that the functional sites in this region may have some regulatory effects on the horn. We screened 16 potential functional sites (Figure 4B, Tables 1, 2 and Supplementary Table S10). Indels4 is located in the upstream region of the promoter, SNP12 and Indels3 are located in the downstream region of the gene, and SNP7, SNP8, SNP9, SNP10, SNP11, Indels1, and Indels2 are located in the intron region. The Fst values of these functional sites are high, indicating that these functional sites differ significantly in horned and hornless sheep populations, which may have some key regulatory effects on horns. The exon region of the *ATP7A* gene has 6 SNP sites. There are 3 synonymously mutated SNP sites, among which the differentiation of SNP1 and SNP3 is not obvious, while the differentiation of SNP2 is large. There were also 3 SNP sites with missense mutations, among which SNP4 (p.A222E) and SNP5 (p.L33Q) differentiation were not obvious, and SNP6 (p.V10I) differentiation was more obvious, which may play a role in certain groups.

**TABLE 1:** Potential SNP functional loci of *ATP7A* gene.

INFO	Mutation type	Position	Fst	Ref	Alt	Ref allele frequency									
						AM	H	BY	CB	OL	PT	STH	T	VT	WZ
SNP1	Synonymous	69092941	0.025	C	T	1	1	1	1	1	0.9	1	1	1	0.9
SNP2	Synonymous	69092872	0.1875	T	C	0.55	0.25	0.7	0.8	0.85	0.5	0.6	0.25	0.6	0.4
SNP3	Synonymous	69075762	0.025	G	A	1	1	1	1	1	0.9	1	1	1	0.9
SNP4	Non-synonymous	69053701	0.025	G	T	1	1	1	1	1	1	1	0.9	1	0.9
SNP5	Non-synonymous	69031423	0.01875	A	T	1	1	1	1	1	1	1	1	0.95	0.9
SNP6	Non-synonymous	69031353	0.125	C	T	0.75	0.9	0.9	1	0.95	1	1	1	0.85	0.9
SNP7	Intronic	69080490	0.59375	A	C	0.25	0	1	1	1	0.45	1	1	0.2	0.1
SNP8	Intronic	69042600	0.6125	C	T	0	0.75	1	1	1	1	1	1	1	0.9
SNP9	Intronic	69027147	0.8625	C	T	0	0	0	1	1	1	1	1	1	0.9
SNP10	Intronic	69009769	0.5125	C	T	1	1	0	0	1	1	0	0	1	0.9
SNP11	Intronic	68976839	0.6875	C	T	1	1	1	0	0	0.5	0	1	0	0
SNP12	Downstream	69112510	0.3125	T	C	0.4	0.25	0.8	0.8	0.85	0.55	0.65	0.3	0.65	0.5

**TABLE 2:** Potential Indels functional loci of *ATP7A* gene.

INFO	Mutation type	Position	Fst	Ref allele frequency									
				AM	H	BY	CB	OL	PT	STH	T	VT	WZ
Indels1	Intronic	69024119	0.51875	1	1	0.05	0	0.15	0.5	0.25	1	1	0.9
Indels2	Intronic	68981839	0.5125	0.55	1	0.25	0.2	0.15	0.45	0.15	0.15	0.4	0.35
Indels3	Downstream	69112032	0.3125	1	0.1	0.9	1	1	1	1	1	1	0
Indels4	Upstream	68950083	0.28125	0.45	0.9	1	1	1	1	1	1	1	0.65

### 3.5 Allele-specific expression in *ATP7A* gene

**FIGURE 5:**
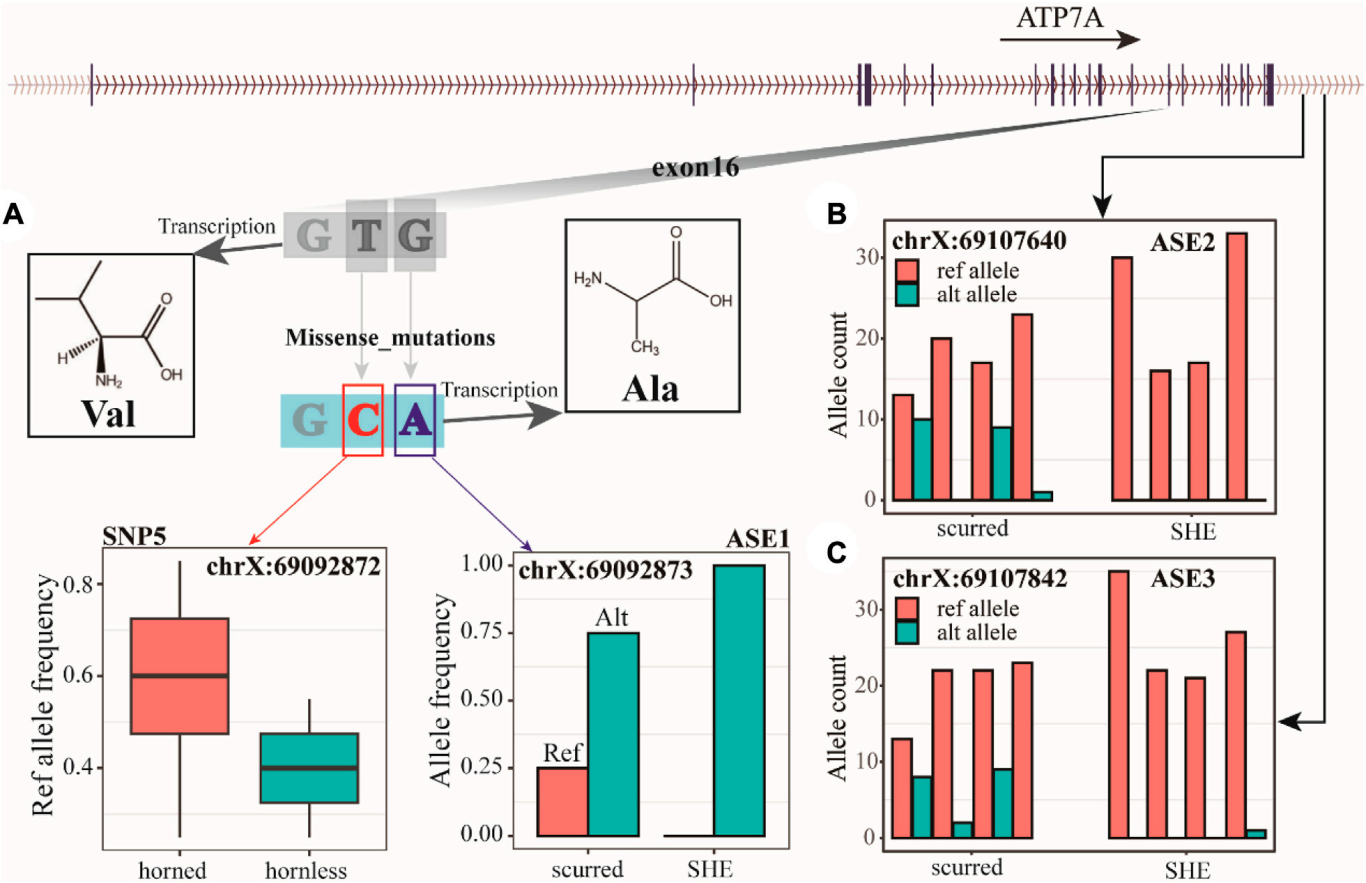
**(A)** The ref allele frequency of SNP5 in horned and hornless populations, the allele frequency of ASE1, and the 1040 amino acid coding of the *ATP7A* gene. **(B)** Count of alleles in ASE2. **(C)** Count of alleles in ASE3.

We identified three AES (allele-specific expression) loci from RNA-seq data, with ASE1 (chrX: 69092873) located in exon 16 of the *ATP7A* gene (Figure 5A), and ASE2 (chrX: 69107640) and ASE3 (chrX: 69107842) located downstream of the *ATP7A* gene (Figure 5B, C). ASE1, ASE2, and ASE3 have both refs (reference) and alt (alternative) alleles in the scurred group. However, ASE1 only has the alt allele in the SHE group, while ASE2 and ASE3 almost only have the ref allele in the SHE group. These three ASE loci may be closely related to horns. Combining WGS and RNA-seq data, we have discovered an interesting phenomenon. ASE1 (chrX: 69092873) and SNP5 (chrX: 69092872) jointly encode amino acid 1040 of ATP7A protein. When these two sites undergo individual mutations, they are both synonymous mutation sites. But when they undergo mutations simultaneously, their encoded Val (valine) will become Ala (alanine). We also found that the ASE3 (chrX: 69107842) was also detected in WGS data. But this site did not show much difference in WGS data.

## 4 Discussion

The horn is a hollow paired structure located on the frontal bone (Nasoori, 2020). The sheep horn consists of two main parts: the outer layer is the keratinized skin tissue, the horn sheath, and the inner layer is the bony horn core (Huang et al., 2017). Mutations in the *ATP7A* gene are associated with Menkes disease and occipital angle syndrome (Beyens et al., 2019; Lenartowicz et al., 2015). It is clinically characterized by connective tissue abnormalities and skeletal changes. Occipital angle syndrome, also known as X-linked knife flaccid disease, begins early in adolescence and is characterized by a wedge-shaped calcified nodule at the junction of the trapezius, sternocleidomastoid muscle, and occipital angle (Kaler and Distasio, 1993), X-ray radiographs reveal occipital bone exostoses and hammer-shaped clavicular heads (Kaler, 2014). This abnormal hyperplasia of bone is very similar to the bone core formation process at the horn base. Therefore, we speculate that the *ATP7A* gene is involved in the formation of the bone core of the horn. From the RNA-seq results, we found that the expression of the *ATP7A* gene in the large horn group (SHE) is higher than that in the small horn group (scurred) at both the gene level and the exon level, suggesting that the *ATP7A* gene may also be involved in the regulation of horn size. On the other hand, the *ATP7A* gene is expressed more in horned cultivars than in hornless breeds, which further proves that *ATP7A* genes may affect the formation and even size of horns. We found that the *ATP7A* gene is specifically highly expressed in soft horns and skin tissues, but almost not expressed in other internal organs, combined with previous studies (Panichsillaphakit et al., 2022), indicating that the *ATP7A* gene is closely related to the function of the skin, may play a role in the keratinization of the skin, and participate in the formation process of horn sheaths. In four animals, humans, cattle, pigs and sheep, the *ATP7A* gene is only highly expressed in the skin of horned animals, sheep. The *ATP7A* gene was highly expressed in the skin of both rams and ewes, and the differences were not significant. We also identified some more interesting amino acid variants that were only present in three horned ruminants, sheep, goats and cattle. Taken together, we suggest that the *ATP7A* gene may be involved in both the formation of the horn bone matrix and the formation of the horn sheath. This suggests that it plays an important role in regulating both the presence and size of the horn.

Based on the analysis of the resequencing data, it was found that to some extent we were able to distinguish between groups with or without horns very clearly. Past studies have found that genes such as RXFP2 and HOXD can control horn phenotype in sheep (Pan et al., 2018; Ren et al., 2016), but these genes only partially explain the phenomenon, suggesting that horned and hornless are not a single point or single gene controlled traits, while the present study found that the *ATP7A* gene may also control the presence or absence of horns to some extent. We identified seven mutation sites of Fst > 0.5 in the intron region of the *ATP7A* gene, and three mutation sites of Fst >0.25 within 10,000 bp upstream and 10,000 bp downstream of the gene. These loci can significantly distinguish the horned and hornless populations and may play a huge role in the presence or absence of horns. We identified six SNP loci located in the exonic region of the *ATP7A* gene, resulting in three synonymous and three missense mutations. SNP6 (p.V10I), is a missense mutation with a high Fst value that may affect the structure and function of ATP7A protein and may play a controlling role in certain populations.

Analyzing the RNA-seq data, we likewise found three more interesting loci. Among them, the SNP located at chrX: 69092873 in the CDS region causes a synonymous mutation, and SNP5 (chrX: 69092872), which co-encode the same amino acid with it, is also a synonymous mutation; both mutations by themselves do not produce changes in the structure of the protein, but when both are mutated at the same time, they change the valine formed by their normal encoding (Val) to alanine (Ala). SNPs located at chrX: 69107640 exhibited allele-specific expression in the scurred and SHE groups, reference alleles could be found in the scurred group, but only alternative alleles existed in the SHE group. The SNP located at chrX: 69107640 also showed the above problem. These two SNP sites regulate the size of the horn. The SNP at chrX: 69107640, which was analyzed in the RNA-seq data, also appeared in the WGS data and was the only SNP site where the RNA-seq results and WGS results intersected.

## 5 Conclusion

This study found that the *ATP7A* gene has the highest expression level in sheep skin tissue and is also highly expressed in soft horn tissue. The *ATP7A* gene may be related to differences in horn size between ram and ewe and the presence or absence of horns. We have identified multiple potential functional sites, including SNP, Indels, and ASE sites, which may be related to horns. We have discovered seven special amino acid sites of ATP7A protein that may be important to the ruminate horn. This study lays the foundation for future research on the functional mechanism of the *ATP7A* gene.

## Data Availability

The datasets presented in this study can be found in online repositories. The names of the repository/repositories and accession number(s) can be found below: https://www.ncbi.nlm.nih.gov/, PRJNA1003277.
